# Dissecting the organ specificity of insecticide resistance candidate genes in *Anopheles gambiae*: known and novel candidate genes

**DOI:** 10.1186/1471-2164-15-1018

**Published:** 2014-11-25

**Authors:** Victoria A Ingham, Christopher M Jones, Patricia Pignatelli, Vasileia Balabanidou, John Vontas, Simon C Wagstaff, Jonathan D Moore, Hilary Ranson

**Affiliations:** Department of Vector Biology, Liverpool School of Tropical Medicine, Pembroke Place, Liverpool, L35QA UK; Systems Biology DTC, University of Warwick, Senate House, Coventry, CV47AL UK; Department of Biology, University of Crete, Vassilika Vouton, 71409 Heraklion, Greece

**Keywords:** Detoxification, Insecticide resistance, Microarray, Mosquito, Transcriptome

## Abstract

**Background:**

The elevated expression of enzymes with insecticide metabolism activity can lead to high levels of insecticide resistance in the malaria vector, *Anopheles gambiae*. In this study, adult female mosquitoes from an insecticide susceptible and resistant strain were dissected into four different body parts. RNA from each of these samples was used in microarray analysis to determine the enrichment patterns of the key detoxification gene families within the mosquito and to identify additional candidate insecticide resistance genes that may have been overlooked in previous experiments on whole organisms.

**Results:**

A general enrichment in the transcription of genes from the four major detoxification gene families (carboxylesterases, glutathione transferases, UDP glucornyltransferases and cytochrome P450s) was observed in the midgut and malpighian tubules. Yet the subset of P450 genes that have previously been implicated in insecticide resistance in *An gambiae*, show a surprisingly varied profile of tissue enrichment, confirmed by qPCR and, for three candidates, by immunostaining. A stringent selection process was used to define a list of 105 genes that are significantly (p ≤0.001) over expressed in body parts from the resistant versus susceptible strain. Over half of these, including all the cytochrome P450s on this list, were identified in previous whole organism comparisons between the strains, but several new candidates were detected, notably from comparisons of the transcriptomes from dissected abdomen integuments.

**Conclusions:**

The use of RNA extracted from the whole organism to identify candidate insecticide resistance genes has a risk of missing candidates if key genes responsible for the phenotype have restricted expression within the body and/or are over expression only in certain tissues. However, as transcription of genes implicated in metabolic resistance to insecticides is not enriched in any one single organ, comparison of the transcriptome of individual dissected body parts cannot be recommended as a preferred means to identify new candidate insecticide resistant genes. Instead the rich data set on *in vivo* sites of transcription should be consulted when designing follow up qPCR validation steps, or for screening known candidates in field populations.

**Electronic supplementary material:**

The online version of this article (doi:10.1186/1471-2164-15-1018) contains supplementary material, which is available to authorized users.

## Background

Insecticides play a vital role in reducing malaria transmission in Africa. An escalation in the use of two very effective tools, indoor residual spraying with insecticides and insecticide treated bednets, has led to impressive reductions in malaria with child death rates halved and more than 3.3 million lives saved since 2000 [1]. Inevitably, as insecticide use has intensified, malaria vectors have developed resistance to these chemicals [2–4]. With just four classes of insecticides available for public health and only the pyrethroids approved for bednet treatment, this poses a major challenge to sustaining and extending recent achievements in malaria reduction.

Numerous studies have attempted to identify the genes responsible for insecticide resistance in the major malaria vectors. One of the most potent mechanisms identified to date is increased activity of enzymes that detoxify insecticides [4–6]. Four enzyme families are known to be associated with insecticide metabolism (carboxylesterases (CCEs), glutathione transferases (GSTs), UDP glucornyltransferases (UGTs) and cytochrome P450s (P450s)) and a number of individual enzymes, most notably from the cytochrome P450 family, have been implicated in conferring resistance to one or more insecticide classes [2, 7–9]. More recently, the importance of interactions between different enzymes and transporters in the insecticide detoxification pathway has been recognised [10, 11]. To dissect these pathways further, and to distinguish members of these large gene families with housekeeping functions from those more likely to have detoxification roles, further information on their sites of expression is required. The first objective of this study was to characterise expression patterns of the key gene families associated with insecticide resistance across the major organs linked to xenobiotic detoxification in insects, with a particular focus on the P450s. Although transcriptomes of many of the key tissues in *Anopheles gambiae* have already been described [12–15], this study used material from a highly insecticide resistant strain and from a susceptible strain to identify genes whose tissue-specific enrichment might be linked to the resistance phenotype.

To date, all comparisons of the transcriptome between insecticide resistant and susceptible malaria vectors have compared gene expression in the whole organism. This approach has the potential to miss candidates. If, for example, expression of a gene is restricted to an organ that contributes only a small proportion of mRNA to the total RNA pool, or differential expression occurs in only one tissue, even large differences in expression between a resistant and susceptible population may not be detectable [16]. Thus the second objective of the study was to compare gene expression in key body parts between an insecticide resistant and susceptible strain of mosquito to identify candidates not immediately apparent in whole organism microarray studies. Adult mosquitoes were dissected into body parts that could be readily separated with minimal risk of contamination, and are suspected to be involved in metabolic resistance.

## Results and discussion

RNA was extracted from three dissected ‘body parts’: the malpighian tubules, the midgut and the abdomen integument (containing the fat body, but also epidermal, neuronal, muscle and oenocyte cells) with the remaining undissected body parts forming a fourth sample group. Each biological replicate consisted of 15–20 adult female mosquitoes from the major African malaria vector, *An. gambiae*. Dissections were performed on an insecticide susceptible strain (N’Gousso) originally from Cameroon [17] and the Tiassalé strain from Cote d’Ivoire, which is highly resistant to all four classes of insecticide [2, 3].

Transcription in these body parts was compared in two ways (i) each body part against the corresponding whole organism for both strains and (ii) resistant against the corresponding susceptible body parts (Additional file 1: Figure S1).

### Gene enrichment in individual body part versus whole organism

**Figure 1:**
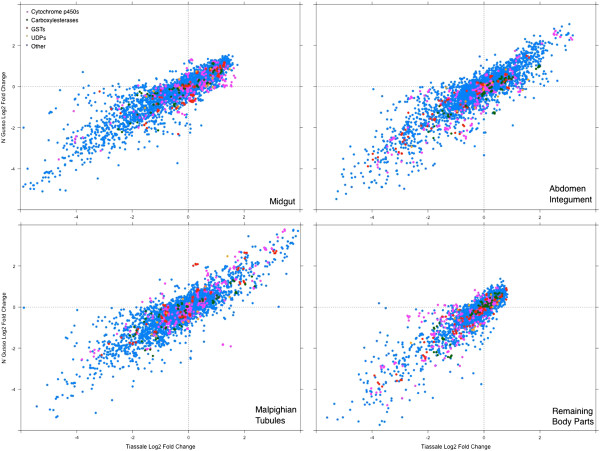
**Gene enrichment in individual body parts in insecticide susceptible and resistant mosquitoes.** Log_2_ fold change in transcription of all probes in individual tissues against the reconstituted whole organism, plotted for Tiassalé (resistant, x) and N’Gousso (susceptible, y). Probes for the four detoxification families are indicated in pink (cytochrome p450s, 393 probes), green (carboxylesterases, 168 probes), red (GSTs, 152 probes) and yellow (UDPs, 26 probes).

Transcripts showing enriched transcription in each of the four body parts were determined using a multiple test correction significance cut-off of p ≤0.05 for both the resistant and susceptible strains. As expected, a clear positive correlation can be observed for local transcription between the resistant and susceptible strains in each body part. This is illustrated in Figure 1, where the vast majority of probes follow a y = x trend.

In all body parts, similar numbers of transcripts showed enriched or depleted transcription compared to the whole organism, with a range of 1.4% to 4.4% of the total probes (Additional file 2: Table S1). The magnitude of change in transcription of individual transcripts in the midgut and remaining body parts is relatively low with no transcripts exceeding log_2_ fold change of 2, compared to the abdomen integument and malpighian tubules where 7 and 42 genes respectively are above log_2_ 3-fold enriched (Figure 1). The full lists of transcripts enriched in each body parts are listed in Additional file 3: Table S2.

The *An. gambiae* genome contains 211 genes encoding four major detoxification families (111 cytochrome P450s, 31 GSTs, 43 CCES (only putatively catalytically active enzymes included) and 26 UGTs), together comprising 1.5% of the probes on the Agilent array [6]. Each of the dissected body parts have an over representation of detoxification transcripts with more members of these gene families overtranscribed relative to the whole organism than found in the ‘remaining’ (undissected body part). Additional file 2: Table S1 shows that 8.5% of the total number of detoxification transcripts are enriched in the malpighian tubules of the resistant strain, 13.7% in the midgut and 7.6% in the abdomen integument. In contrast, the ‘remaining’ (undissected body part) show a depletion of detoxification transcripts in the overtranscribed subset when compared to the whole organism. These data reinforce the importance of the selected body parts in xenobiotic detoxification.

**Figure 2:**
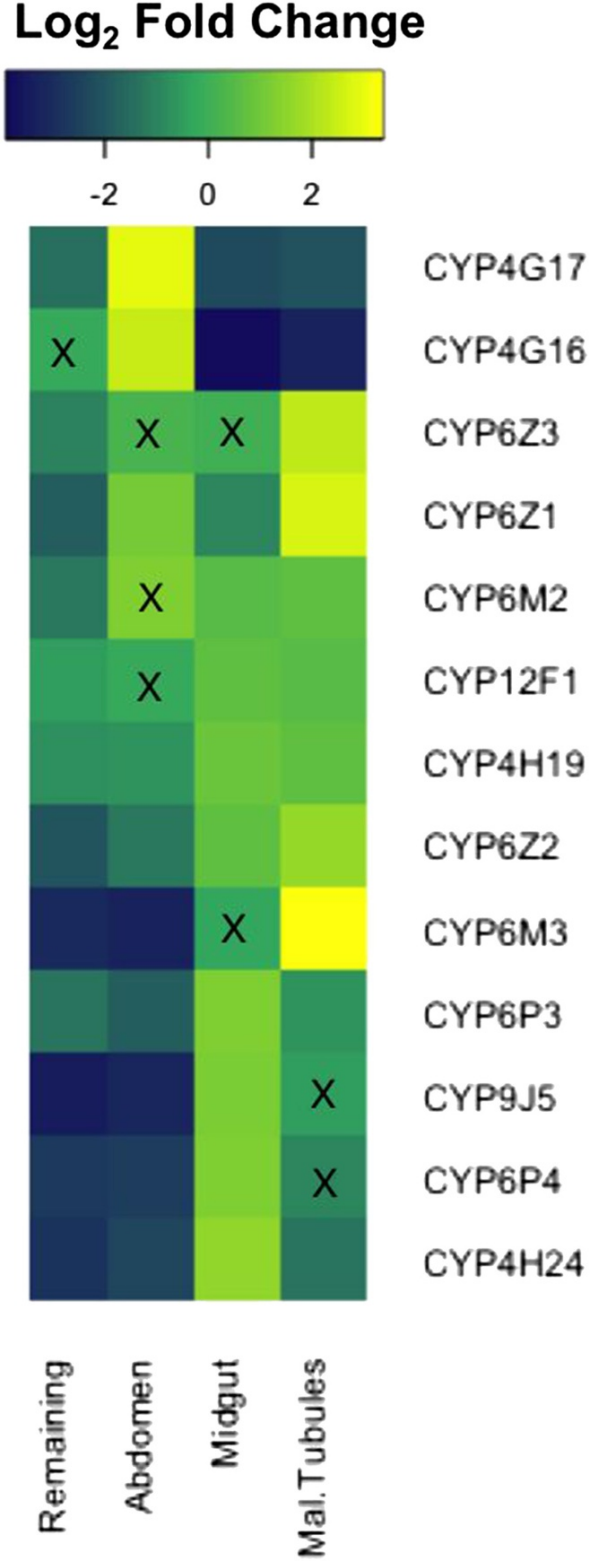
**Local expression of cytochrome P450s linked to insecticide resistance.** Heatmap showing the log_2_ fold change of a subset of cytochrome p450s (implicated in insecticide resistance in previous studies) in different body parts of the Tiassalé strain. Crosses indicate non-significance (p >0.05).

The cytochrome P450 family has been most strongly linked with insecticide resistance in *Anopheles* mosquitoes, with several enzymes capable of detoxifying insecticides from more than one class [2, 8]. Identifying the primary sites of transcription of this enzyme family will aid prediction of function [18] and help identify the key organs largely responsible for insecticide detoxification in resistant mosquitoes. Within the P450 gene family, body part enrichment shows some relationship with the gene tree clustering (Additional file 4: Figure S2) with the CYP9J and 6P families largely enriched in the midgut, the CYP4Gs, 6Ys and 325Cs enriched in the abdomen integument and the CYP6Z family enriched in the malpighian tubules. The diversity in enrichment patterns within this gene family led us to look specifically at thirteen cytochrome P450s that had been implicated in insecticide resistance to see if these were enriched in a particular body part (Figure 2). The criteria for inclusion of these P450s as candidate insecticide resistant genes was that they had been found to be significantly over expressed in pyrethroid and/or DDT resistant *An. gambiae* populations in more than one independent study. Four of these transcripts were significantly (p < 0.05) and highly (Log_2_ Fold Change >1.5) enriched in the malpighian tubules (*CYP6M3*, *CYP6Z1, CYP6Z2* and *CYP6Z3*) compared to whole organism and one was enriched in the midgut and two in the abdomen integument (CYP4H24 and *CYP4G16/CYP4G17* respectively). The remaining 5 showed no significant tissue enrichment.

**Figure 3:**
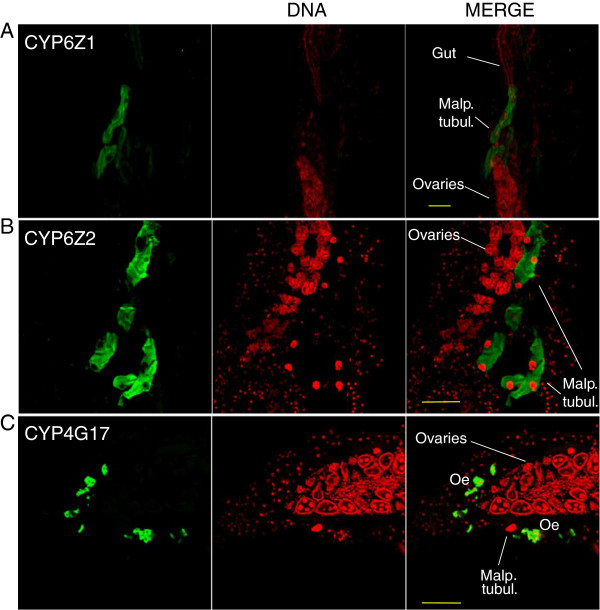
**Immunohistochemical stainings of cytochrome P450s associated with pyrethroid resistance.** Longitudinal sections from resistant mosquito (Tiassalé) specimens immunostained with **A)** a-CYP6Z1, **B)** a-CYP6Z2 and **C)** a-CYP4G17 specific antibodies (left panel, green color). The middle panel shows the same sections stained red using the nucleic acid stain TOPRO. The merged immunohistochemical staining (P450 stainings and nuclei) appears in the right column. Bar scale (yellow line): 100 nm.

The enrichment of a subset of seven cytochrome P450s in particular body parts was confirmed by qPCR, although for two of these midgut enriched transcripts, a much greater over expression was observed by qPCR than for microarray (Additional file 5: Figure S3) (A level of discordance between qPCR and microarray is frequently observed [19] (but it in the current study, the direction of change was consistent between the two methodologies for all seven genes). Antibodies were available for three cytochrome P450s, which were used to confirm the major sites of transcription within the abdomen integument of the Tiassalé resistant strain. In agreement with microarray transcription data, CYP6Z1 and CYP6Z2 were detected in the malpighian tubules of resistant mosquitoes, whilst CYP4G17, identified as enriched in the abdomen integument by both microarray and qPCR, was found only in the oenocytes, a cellular layer located under the abdomen cuticle (Figure 3). A CYP4G from *Drosophila melanogaster*, CYP4G1, has also been shown to be highly enriched in oenocyte cells; this *Drosophila* enzyme catalyses a key step in the formation of cuticular hydrocarbons [20]. The potential role of over expression of 4G17, and its paralogue 4G16, in altering the cuticular structure in insecticide resistant mosquitoes is currently under investigation.

### A new list of candidate insecticide resistance genes from the body part specific arrays

**Figure 4:**
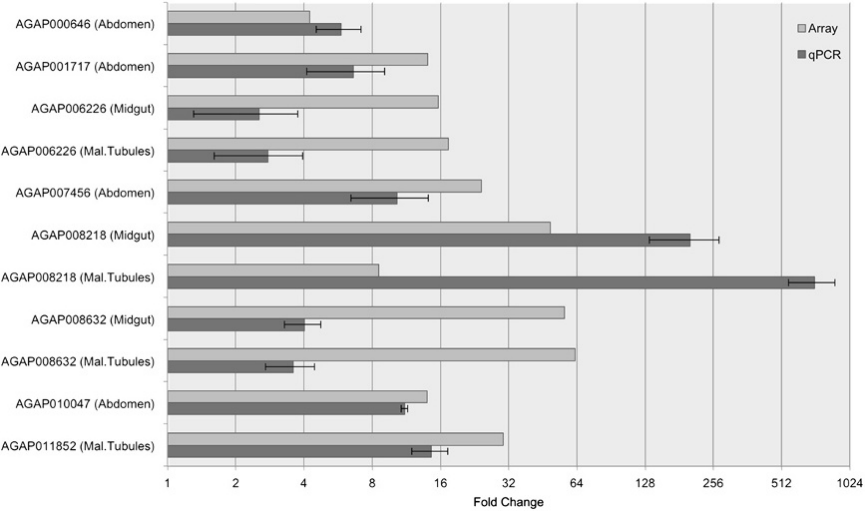
**Validation of selection of genes from the candidate list by qPCR.** Log_2_ fold change from qPCR data and array data of the direct comparison between the resistant and susceptible populations [29, 30]. Genes were selected from the ‘stringent candidate gene list’ consisting of probes enriched in the resistant versus susceptible strain in one or more dissected body parts. Standard error bars are shown.

The two strains used in this study originate from sites separated by approximately 2,500 km, so substantial variation in gene transcription between them is to be expected, regardless of the difference in their insecticide resistance profile. Thus caution must be applied when correlating gene transcription levels with the resistance phenotype. However, with a goal of identifying further candidate insecticide resistance genes from the direct comparisons between dissected body parts from the resistant and susceptible strain for further functional validation, we applied a stringent selection process to derive a new gene list. This involved selecting only genes detected as enriched in body parts from Tiassalé versus N’Gousso via both the limma and GaGa methods with an adjusted p value ≤0.001 and setting a cut off of 1.4-fold differential expression (see Methods section). This identified a list of 134 transcripts, representing 105 genes transcribed at higher levels in the Tiassalé strain and 16 genes with higher transcription in the susceptible N’Gousso strain (Additional file 6: Table S3). Eleven of these transcripts were selected for qPCR validation yielding a positive correlation with the array (Pearson’s correlation (r = 0.870)) (Figure 4).

All of the 9 cytochrome P450s (*CYP6P2*, *P3* and *P4*, *CYP6Z2, Z3 CYP4H17*, *CYP4K2, CYP4C35* and *CYP4G16*) on this candidate list were also detected in the direct whole comparisons of the whole organism transcriptomes between these strains [3 and C. Strode, unpublished data]. Indeed the majority (89) of the 134 transcripts on our candidate list derived from the comparisons of dissected body parts were also detected in comparisons of the entire transcriptome between the two strains. Nevertheless, 22 (16.4%) would have been missed using whole organism arrays and 23 (17.2%) of the genes are regulated in the opposite direction between the whole organism and dissected body part comparisons. Of the 22 transcripts not detected in strain comparisons at the whole organism level, the majority (13) were detected from the abdomen integument (Table 1). Abdomen integuments from other resistant strains of *An. gambiae* are being dissected to search for further supporting evidence for a role of these transcripts in conferring resistance prior to follow up functional analysis.

**Table 1:** **Genes showing the greatest differential expression between resistant and susceptible strains in specific body parts, but which were not identified in whole transcriptome comparisons**

AGAP identifier	Description	Tissue	Log_2_fold change
AGAP006710	Chymotrypsin-1	Midgut	7.20
AGAP006741	Putative Tight Junction Associated Protein	Abdomen Integument	2.33
AGAP001769	Beat Protein	Abdomen Integument	2.27
AGAP007650	GADD45 (Growth Arrest and DNA Damage Inducible Protein)	Abdomen Integument	2.15
AGAP000717	Monocarboxylate Transporter	Midgut	1.52
AGAP005563	Sugar Transporter	Abdomen Integument	1.91
AGAP009521	Ankyrin Erythrocytic	Abdomen Integument	1.86
AGAP007879	Steroid Dehydrogenase	Midgut	1.74
AGAP013219	Elongase	Abdomen Integument	1.70
AGAP007301	Conserved hypothetical protein	Malpighian Tubules and Midgut	1.61
AGAP001649	Glucosylceramidase	Abdomen Integument	1.11
AGAP006358	TTC27	Abdomen Integument	1.05
AGAP002517	Unknown	Abdomen Integument	0.95
AGAP007691	Serpin 18	Abdomen Integument	0.92(-RC)
			0.87(-RB)
AGAP005651	Cytoplasmic tRNA 2-thiolation protein	Abdomen Integument	0.80
AGAP004099	Conserved Hypothetical Protein	Abdomen Integument	0.60
AGAP005334	C-Type Lectin	Midgut	-3.62
AGAP003271	Anexin B10B	Midgut	-1.87
AGAP002752	DNAJ Homolog	Remaining Body Parts	-1.10

Splice variants, as predicted by VectorBase, are labelled with variations of -Rx.

## Conclusions

Microarrays are widely used to identify insecticide resistance mechanisms in mosquito populations [3, 9, 21, 22]. However, all of these studies have used whole-organism RNA as a template, which may dilute or distort the final level of transcript expression detectable. By dissecting some of the major body parts involved in xenobiotic detoxification from two strains of *An. gambiae*, differing in their resistance phenotype we have been able to simultaneously identify the local expression profiles of known insecticide resistance candidates and compare transcription between the two strains within individual body parts.

This rich data set will be useful for establishing pathways of detoxification as genes catalysing the three classic classes of drug metabolism (oxidation, conjugation and excretion) [23] would be expected to be co-regulated. Pyrethroid mimetic activity–based probes, used to detect pyrethroid metabolising enzymes in the rat liver, identified a potential network of drug metabolising enzymes from multiple families involved in pyrethroid metabolism [10]. Applying the same approach to insecticide resistant mosquitoes and using the data on local transcription from the current data set, will help unravel the pathways of insecticide metabolism selected for by intensive use of pyrethroids.

Although potential new insecticide resistance candidates have emerged from this study, it is encouraging that the majority of candidate insecticide resistant transcripts identified from direct comparison of the transcriptomes of dissected body part were also detected in the whole organisms comparisons. No single body part emerged as the key site of overtranscription of putative insecticide resistance genes in this study and it is therefore recommended that, unless resources enable a more comprehensive study design involving multiple dissected tissues, transcriptional approaches to identify candidate insecticide resistance transcripts continue to use the whole body transcriptome. Nevertheless this data set on local sites of transcription should be consulted when designing follow up qPCR validation steps, or for screening known candidates in field populations.

## Methods

### Mosquito rearing conditions

The *An. gambiae* used in these experiments were all reared under standard insectary conditions at 27°C and 70-80% humidity under a 16:8 hour photoperiod. The N’Gousso strain is originally from Cameroon and is susceptible to all classes of insecticide [17]. N’Guosso is the M molecular form of *A gambia*e, recently re-classified as a separate species, *Ano coluzzi*[24]. In contrast, the Tiassalé strain from Côte D’Ivoire is resistant to all classes of insecticide [2, 3]. This strain was colonised from the field site in 2012 and is a mixture of the M and S molecular forms. At the time of the study, the LD_50_ for the Tiassalé strain was 68 and 81 fold higher than the corresponding value for the N’Gousso strain for permethrin and deltamethrin respectively. Further details of the resistance profile of this strain are contained within references by Edi et al. [2, 3].

### Microarray experiments

RNA was extracted from three dissected body parts: the malpighian tubules, the midgut and the abdomen integument (containing the fat body but also epidermal, neuronal, muscle and oenocyte cells) with the remaining undissected body parts forming a fourth sample group. Mosquitoes were collected between the hours of 8AM and 2 pm and dissected immediately on a CO_2_ block. Post dissection, each body part was added to extraction buffer from the PicoPure RNA extraction kit, heated for 30 minutes at 42°C and frozen at -80°C as per manufacturers instructions. Each biological replicate for each strain consisted of RNA, extracted using PicoPure RNA Isolation kit (Arcturus), from 12 3–5 day old non-blood fed, presumed mated females. The quantity and quality of the RNA was assessed using a nanodrop spectrophotometer (Nanodrop Technologies UK) and Bioanalyser (Agilent) respectively. Four biological replicates were prepared for each body parts per strain. RNA from the four dissections was pooled according to the proportion of RNA extracted from each body part to reconstitute the ‘whole organism’ (7%, 6%, 24% and 63% RNA from abdomen integument, malpighian tubules, midgut, and remaining material respectively). The use of a reconstituted reference sample minimised potential sources of bias that could have arisen from circadian changes in gene expression and changes in the proportion of the M or S molecular form in the different biological replicates. 100 ng of RNA was amplified and labelled with Cy3 and Cy5, using the Two colour low input Quick Amp labelling kit (Agilent) following the manufacturers instructions. Samples were then purified (Qiagen) with the cRNA yield and quality assessed using the nano-drop and Bioanalyser respectively. RNA from each Tiassalé body part was competitively hybridised with the respective N’Gousso body part, as well as each body part from the resistant and susceptible strain being compared to the re-constituted whole organism (Additional file 1: Figure S1). Dye swaps were performed on two out of four technical replicates for each array, to correct for dye bias.

Labelled cRNAs were hybridised to the whole genome 8×15k *An gambiae* array (ArrayExpress accession number A-MEXP-2211). Microarray hybridisation, washing and scanning were performed according to previously described protocols [8].

### Microarray analysis

The resulting data were analysed using R. Within-array normalisation was carried out by Loess, and between array normalisation by Aquantile. Signals were corrected for dye automatically. The limma package [25] was used to fit linear models to the normalised data. In the case of complete loop designs a design matrix was used to infer the appropriate contrast matrices for each array. All parameters used were default. A bespoke pipeline using the GaGa package [26] was used to fit gamma-gamma models of variation to normalised corrected signals, in order to assign probes to one of two patterns of expression X equals Y or X does not equal Y, where X represents the resistant population arrays and Y the susceptible arrays. These data were subsequently used to assess enrichment in each expression pattern, through GO term analysis using the TopGO package [27]. A standard FDR adjusted p value cut off of p ≤0.05 was applied to all data describing localisation of detoxification candidates. A second stringent selection method was used to reduce the probe list based on previously published methodology [19], requiring that the following criteria were met: adjusted p-value ≤0.001, raw fluorescence intensity > median, and Tiassalé vs. N’Gousso ±0.485 Log_2_ fold change between the strains. All candidates selected also demonstrated a positive GaGa analysis fold change, indicative of higher transcript localisation in the resistant tissue to the susceptible, thereby utilising all available array data.

### RT-qPCR

RNA (4 μg) from each biological replicate was reverse transcribed using Oligo dT (Invitrogen) and Superscript III (Invitrogen) according to manufacturers instructions. Quantitative real-time PCR was performed using SYBR Green Supermix III (Applied Biosystems) using an MX3005 and the associated MxPro software (Agilent). Primer Blast (NCBI) [28] was used to design primer pairs (Additional file 7: Table S4). Where possible, primers were designed to span an exon junction but this was not possible for six of the P450 genes (*CYP325A1*, *CYP6P3*, *CYP4G17*, *CYP6Z3*, *CYP12F2* and *CYP6Z2*) due to the high degree of polymorphisms in their DNA sequence. Each 20 μl reaction contained 10 μl SYBR Green Supermix, 0.3 μM of each primer and 1 μl of 1:10 diluted cDNA. Standard curves were produced using whole N’Gousso cDNA, in 1, 1:5, 1:25, 1:125 dilutions, (48.3 ng/μl to 0.386 ng/μl). qPCR was performed with the following conditions: 3 minutes at 95°C, with 40 cycles of 10 seconds at 95°C and 10 seconds at 60°C. All amplification efficiencies of designed primers were within acceptable range (90-120%), following MIQE guidelines [29].

### Preparation of antibodies

Fragments encoding unique peptides for *CYP6Z1* and *CYP4G17* were cloned into the pET 16b vector. Upon expression, the resulting His-tagged peptide was purified to homogeneity by Ni-NTA affinity chromatography and used to raise rabbit polyclonal antibodies. The CYP6Z1 peptide sequence was: VALRDLNNPDSFINNIRTAGVFLCPGLLKFTGINSLSPPMKKFTTEVISSHLHQRETGQVTRKDFIQMLTDLRRKAGSSGEETLTDA and the CYP4G17 peptide: KRQLKIHLRLDPLFNLTGVKKEQERLLQIIHGLTRKVVREKKQLYERQMAEGKMPSPSLTEIIGKEEKPGEGQLGGSPAFISQ. The antibody for CYP6Z2 was a gift from Dr Mark Paine (LSTM, UK). Rabbit antibodies to CYP6Z2 were prepared to the C terminal peptide sequence MRIDHRK by Moravian Biotechnology, Brno, Czech Republic.

### Immunofluorescence and microscopy

Female mosquitoes (3–5 days old) were fixed in cold solution of 4% PFA (methanol free, Thermo scientific) in phosphate-buffered saline (PBS) for 4 h and then were cryo-protected in 30% sucrose/PBS at 4°C for 12 h. Finally, mosquitoes were immobilized in O.C.T. (Tissue-Tek, SAKURA) and stored at -80°C until use.

Immunofluorescent analysis, followed by confocal microscopy, was performed to longitudinal sections of frozen pre-fixed mosquito specimens. More detailed, 10 mm sections, obtained in Leitz kryostat 1720 digital, were washed (3 × 5 min) with 0,05% Tween in PBS and blocked for 3 h in blocking solution (1% Fetal Bovine Serum, biosera, in 0,05% Triton/PBS). Then, the sections were stained with rabbit primary antibodies in 1/500 dilution, followed by goat anti-rabbit (Alexa Fluor 488, Molecular Probes) (1/1000) that gave the green colour. Also To-PRO 3-Iodide (Molecular Probes), which stains DNA specifically (red colour), was used, after RNAse A treatment. As controls, pre-immune serums (in 1/500 dilutions) and anti-rabbit (Alexa Fluor 488, 1/1000) were tested, in parallel with a-P450’s to check specificity of each primary antibody. Finally images were obtained on Leica TCS-NT Laser Scanning microscope using the 40-objective.

### Supporting information

The data sets supporting the results of this article are included within the article and its additional files. All microarray datasets are MIAME compliant [30] and deposited in ArrayExpress and VectorBase (Accession numbers E-MTAB-2808).

## Electronic supplementary material

Additional file 1: Figure S1: Schematic of design of microarray experiment. a. Sample vs reference design, for each of the susceptible lab population N’Gousso and the resistant population Tiassalé b. Resistant (T) vs Susceptible (N) design, for each individual tissue. (PNG 332 KB)

Additional file 2: Table S1: Overview of probes over or under transcribed in each body part for both the resistant and susceptible strains when compared to the whole organism. The local transcription of probes in each of the two mosquito strains is expressed as total number of probes over or under transcribed in a particular body part, compared to the reconstituted whole organism, as a percentage of the total probes on the array. For genes that were represented by multiple probes, an average of all the probes was used. (XLSX 56 KB)

Additional file 3: Table S2: Genes significantly enriched or depleted in one or more body part compared to the whole organism. AGAP identifiers, description and the body part in which the gene is enriched with log_2_ fold change (relative to reconstituted whole transcriptome) indicated for each strain. Only probes outside the 95% intervals are listed. Blue indicates cytochrome p450s, green GSTs, purple COEs, orange UDPs and teal carboxylesterases. (XLS 253 KB)

Additional file 4: Table S3: Probe list from stringent analysis of direct transcriptome comparisons of dissected body parts from susceptible and resistant strains. AGAP identifier, description, body part that the probe fits the stringent selection criteria, whole organism array log_2_ fold change, resistant vs susceptible array log_2_ fold change, GaGa log_2_ fold change and log_2_ body part qPCR results for given tissue. qPCR validation has been performed on several candidates. Cells are coloured with a gradient dependent upon the directionality of the fold change, down regulated transcripts are indicated in red and up regulated in green. Sheets for both up regulated and down regulated genes are present. (PNG 151 KB)

Additional file 5: Figure S2: Local expression of all Cytochrome p450s, following a phylogenetic dendrogam. Full length protein sequence alignment and neighbour joining tree as computed on MEGA5 decorated with local log_2_ transcription of Tiassalé cytochrome p450s. (PNG 62 KB)

Additional file 6: Figure S3: qPCR validation of body part enrichment of cytochrome P450s. Difference in transcription levels in Tiassalé RNA from individual dissected body parts, compared to the reconstituted whole, were measured by qPCR for six P450 genes and compared to the data obtained from the microarray. Data represent log_2_ fold change with associated standard error bars. (XLSX 53 KB)

Additional file 7: Table S4: qPCR primer list. Forward and reverse primers used for all qPCR reactions. All primer products are between 80 and 150 base pairs and follow MIQE guidelines. (XLSX 46 KB)

## Abbreviations

cDNA: Complementary DNA
cRNA: Complementary RNA
FDR: False discovery rate
GO: Gene ontology
mRNA: Messenger RNA
P450s: Cytochrome p450s
PBS: Phosphate-buffered saline
qPCR: Quantitative PCR
UDP: Uridine disphosphate.
