# Supplementary figures and images for: Dissecting the organ specificity of insecticide resistance candidate genes in Anopheles gambiae: known and novel candidate genes
Source: BMC Genomics. 2014 Nov 25;15(1):1018. doi: 10.1186/1471-2164-15-1018 (PMC4256904; doi:10.1186/1471-2164-15-1018)

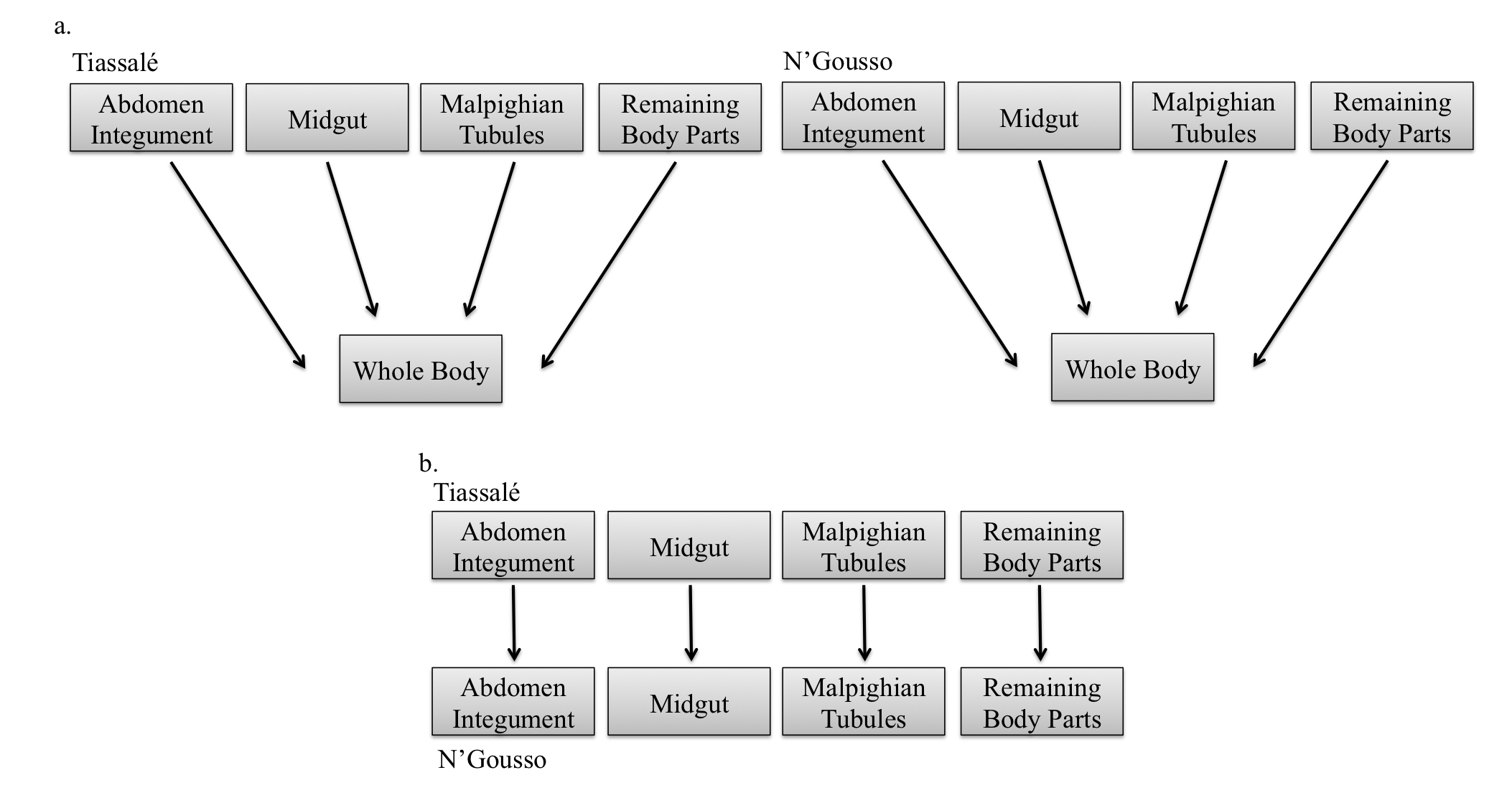

Supplement: Supplementary file 1 — Additional file 1: Figure S1: Schematic of design of microarray experiment. a. Sample vs reference design, for each of the susceptible lab population N’Gousso and the resistant population Tiassalé b. Resistant (T) vs Susceptible (N) design, for each individual tissue. (PNG 332 KB) [file 12864_2014_6715_MOESM1_ESM.png]

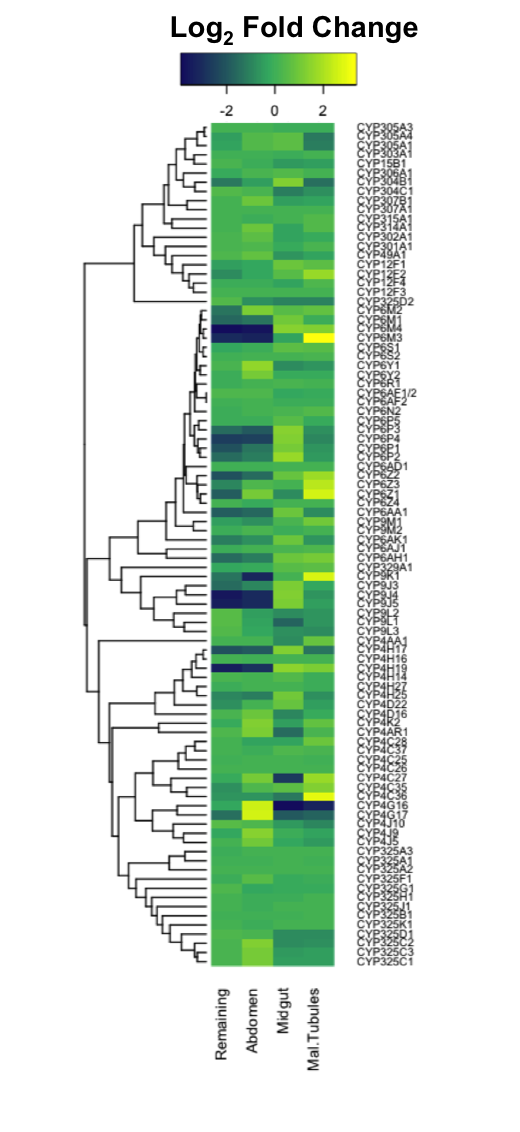

Supplement: Supplementary file 4 — Additional file 4: Table S3: Probe list from stringent analysis of direct transcriptome comparisons of dissected body parts from susceptible and resistant strains. AGAP identifier, description, body part that the probe fits the stringent selection criteria, whole organism array log2 fold change, resistant vs susceptible array log2 fold change, GaGa log2 fold change and log2 body part qPCR results for given tissue. qPCR validation has been performed on several candidates. Cells are coloured with a gradient dependent upon the directionality of the fold change, down regulated transcripts are indicated in red and up regulated in green. Sheets for both up regulated and down regulated genes are present. (PNG 151 KB) [file 12864_2014_6715_MOESM4_ESM.png]

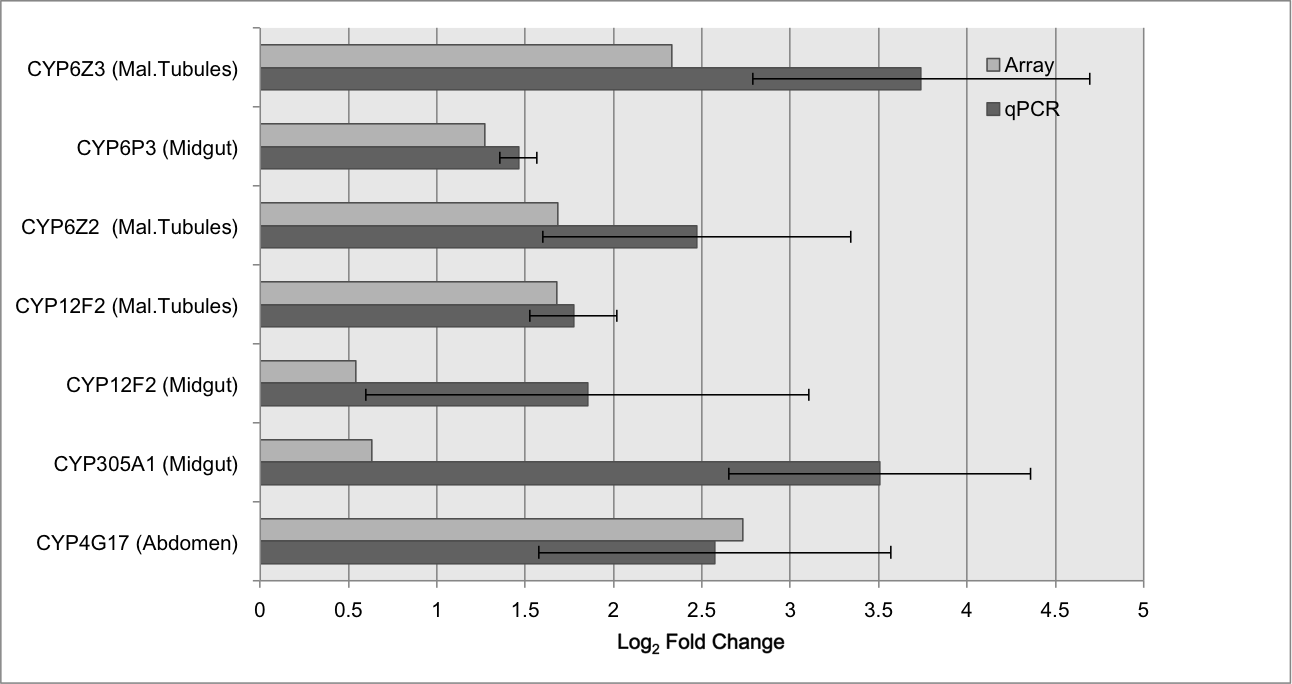

Supplement: Supplementary file 5 — Additional file 5: Figure S2: Local expression of all Cytochrome p450s, following a phylogenetic dendrogam. Full length protein sequence alignment and neighbour joining tree as computed on MEGA5 decorated with local log2 transcription of Tiassalé cytochrome p450s. (PNG 62 KB) [file 12864_2014_6715_MOESM5_ESM.png]
